# Supplementary material for: Coronary Microvascular Dysfunction in Acute Cholestasis-Induced Liver Injury
Source: Biomedicines. 2024 Apr 16;12(4):876. doi: 10.3390/biomedicines12040876 (PMC11048529; doi:10.3390/biomedicines12040876)
Supplement: Supplementary file 1 [file biomedicines-12-00876-s001.zip › biomedicines-2952927-supplementary.pdf]

Supplement Table S1. Echocardiographic parameters before study initiation. Beats per minute (bpm); bile-duct digation (BDL).

parameter	control (mean±SD)	BDL (mean±SD)	p-value	
**cardiac function**				
heart rate (bpm)	341.8±14.1	343±37.5	0.93	ns
n	9	28		
stroke volume (µl)	341.2±55.2	355±55.7	0.52	ns
n	9	28		
ejection fraction (%)	59.1±5.8	59.8±5.7	0.73	ns
n	9	28		
end-diastolic volume (µl)	586.5±69.67	584.7±70.43	0.95	ns
n	9	28		
cardiac output (ml/min)	116.9±22.2	120.9±19.2	0.61	ns
n	9	28		
**coronary artery Pulsed-wave Doppler**				
diastolic peak velocity (mm/s)	679.8±212.7	642.6±160.7	0.58	ns
n	9	28		
systolic peak velocity (mm/s)	220±58.3	186±50.8	0.1	ns
n	9	28		
velocity time integral (mm)	35.1±12.4	32.5±9.2	0.51	ns
n	9	28		

Before induction of acute live injury in rats by surgical bile duct ligation (BDL) an echocardiographic evaluation was performed. The evaluation included standard myocardial function parameter as stroke volume (SV), ejection fraction (EF), end-diastolic volume (VED) and cardiac output (CO) and a Pulsed-wave Doppler assessment of the left coronary artery. Diastolic and systolic peak velocity (DPV; SPV) as well as velocity time integral (VTI) were determined from the velocity time curve.

Data were analyzed using a two-sided t-test or a non-parametric test where appropriate. $P<0.05$ was considered significant and the data are presented as mean ± standard deviation (SD).
